# Regulation of T Cell Activities in Rheumatoid Arthritis by the Novel Fusion Protein IgD-Fc-Ig

**DOI:** 10.3389/fimmu.2020.00755

**Published:** 2020-05-15

**Authors:** Jing Zhang, Xiaoxi Hu, Xiaojie Dong, Wensheng Chen, Lingling Zhang, Yan Chang, Yujing Wu, Wei Wei

**Affiliations:** Key Laboratory of Anti-inflammatory and Immune Medicine, Ministry of Education, Anhui Collaborative Innovation Center of Anti-inflammatory and Immune Medicine, Institute of Clinical Pharmacology, Anhui Medical University, Hefei, China

**Keywords:** immunoglobulin D, rheumatoid arthritis, collagen-induced arthritis, IgD-Fc-Ig fusion protein, T cells

## Abstract

Rheumatoid arthritis (RA) is an autoimmune disease characterized by chronic inflammation and T cell hyper-activation. Emerging evidence has shown that the stimulation of immunoglobulin D (IgD) induces T cell activation and may contribute to disease pathogenesis. In this study, the sIgD concentrations were positively associated with disease activity score in 28 joints (DAS28) and anti-cyclic citrullinated peptide (anti-CCP) in RA. We demonstrated that IgD-Fc-Ig (composed of human IgD Fc domain and IgG_1_ Fc domain, obtained through prokaryotic protein expression and chromatography purification) effectively inhibited the activation and proliferation of T cells in healthy controls and PBMCs in RA patients stimulated by IgD, recovered the Th17/Treg cell subset balance, and downregulated p-Lck and p-ZAP70 expression. Moreover, *in vivo*, IgD-Fc-Ig decreased the swollen joint counts and arthritis indices in mice with collagen-induced arthritis (CIA), and ameliorated histopathological changes in joint and spleen tissue. It also downregulated thymocyte proliferation and reduced the percentage of helper T cells (Th) and CD154^+^ T cells, reversed the imbalance of Th1/Th2 and Th17/Treg cell subsets, reduced cytokine and chemokine levels, and inhibited p-Lck and p-ZAP70 expression. Our data suggest that IgD-Fc-Ig fusion protein regulates T cell activity in RA. These findings have potential implications for IgD-targeted strategies to treat IgD-associated RA.

## Introduction

Rheumatoid arthritis (RA) is the most common chronic autoimmune disease and induces mobility restriction, which is also characterized by chronic inflammation, synovium hyperplasia, joint destruction, and T cell hyper-activation (1). Approximately 1% of the global population suffers from RA (2, 3). Although the precise etiology of RA remains unclear, studies have reported CD4^+^ T cell infiltration in affected joints (4). Thus, inhibition of T cell hyper-activation has been identified as a potential therapeutic target for RA (5). Currently, disease-modifying antirheumatic drugs (DMARDs) and non-steroidal anti-inflammatory drugs (NSAIDs) are widely employed to treat RA (6, 7). Furthermore, newly emerging treatments targeting inflammatory cytokines, such as TNF-α production co-stimulated by B cells and T-cell co-stimulation, have also been used to treat RA; however, many patients do not respond well to this therapy (8). This treatment may also cause serious adverse reactions including immunosuppression, leading to increased risk of infections and malignant tumors. Therefore, exploration and identification of novel approaches and highly selective drugs for further RA treatment is necessary.

Immunoglobulin D is a membrane receptor-bound protein (mIgD) primarily expressed in B cells of both humans and mice. It is also moderately expressed in the secreted form (sIgD) as an antibody (9–11). Engagement of basophil-bound IgD by antigen would then trigger basophil release of IL-4, which may amplify humoral immunity (12). Previous reports suggest that IgD receptor (IgDR) on T cells may regulate IgD expression (13–15). T cell abnormalities, such as exaggerated CD4^+^ T cell activity, are frequently found in patients with RA (16), suggesting that abnormal T cell activation may contribute to disease pathogenesis. Our previous studies have shown that serum levels of sIgD and IgDR expression on T/B cells in RA patients were higher than in healthy controls (17, 18). IgD may also be involved in enhancing the proliferation of peripheral blood mononuclear cells (PBMCs) in RA patients, inducing T cell activation and increasing IgDR expression (19). The IgD-induced IgDR expression levels in T cells was found to be higher than in B cells both in RA patients and healthy controls (17), which shifted our focus to IgD function in T cells, specifically in RA. Thus, investigating IgD-IgDR interactions may offer a potential therapeutic strategy that can be used to treat overexpressed IgD in RA. To block interactions between IgD-IgDR, the target gene encoding the human IgD Fc domain and IgG_1_ Fc domain was cloned using PCR. The recombinant protein was then obtained through prokaryotic protein expression and chromatography purification. Lastly, IgD-Fc-Ig fusion protein was synthesized, and a patent license was acquired in China (No:201510600762X).

In this study, we sought to determine the effects of IgD and IgD-Fc-Ig on PBMCs and T cell function in RA patients and healthy controls *in vitro*, while also further describing the therapeutic effect and underlying mechanism of IgD-Fc-Ig by using collagen-induced arthritis (CIA) mice.

## Materials and Methods

### Patients

The study protocol was carried out in accordance with the Declaration of Helsinki and approved by the Ethics Committee of Anhui Medical University (No. 20160119, 20160095). Blood samples from healthy volunteers and RA patients were collected from the First Affiliated Hospital, Anhui Medical University. Written informed consent was obtained from each donor.

### Clinical Characteristics of RA Patients and Human Plasma IgD Detection

Medical history, baseline characteristics, and demographic data were recorded and physical examination was performed for each patient at the beginning of the study. The levels of sIgD were determined in serum samples using the ELISA method according to the manufacturer's instructions. The clinical indexes of 33 RA patients were collected, including gender, age, RF (rheumatoid factor), CRP (C-reactive protein), ESR (erythrocyte sedimentation rate), and anti-CCP (anti-cyclic citrullinated peptide).

### Reagents

Human IgD was purchased from Abcam (Cambridge, MA, USA). Anti-CD3 and CD28 antibodies were provided by T&L Biological Technology (Beijing, China). A770041 was purchased from Axon Medchem (Groningen, Netherlands). rhTNFR:Fc fusion protein was purchased from Guojian Pharmaceutical Company (Shanghai, China). Anti-mouse IgD antibodies were purchased from eBioscience (San Diego, CA, USA). Anti-human CD69-PE-cy5, CD154-PE, CD4-FITC, CD25-APC, IL-4-APC, IFN-γ-PE-cy7, IL-17-PE, and FoxP3-PE, along with anti-mouse CD3e-PerCP-Cy^TM^5.5, CD4-FITC, CD25-APC, CD154-PE, IFN-γ-PerCP-Cy^TM^5.5, IL4-APC, IL-17-PE, and Foxp3-PE antibodies were provided by BD Pharmingen (San Diego, CA, USA). Anti-Lck, anti-phospho-ZAP70, anti-phospho-Lck, anti-ZAP70, and anti-β-actin were purchased from Affinity Biosciences, Sigma and Abcam.

### Purification of IgD-Fc-Ig Fusion Protein

*IgD-Fc* and *IgG*_1_*-Fc* genes were amplified by RT-PCR, then were connected by overlap PCR method to get *IgD-Fc-Ig* target gene. *IgD-Fc-Ig* target gene was inserted in the prokaryotic expression vector: PET28a(+) to get PET28a (+)/IgD-Fc-Ig plasmid. Then the plasmid was transformated into BL21-DE3 E. coli. IPTG (Isopropyl β D thiogalactopyranoside) were used to induce the expression of the target protein. Affinity and molecular sieve chromatography were used to purify the expression product. His-tag affinity chromatography and ion exchange column were used for purification and endotoxin removal. Coomassie Brilliant Blue staining was applied for purity detection. IgD-Fc-Ig can be applied for study with a purity of more than 90%.

### Competitive Binding Assay of IgDR on the Surface of CD4^+^ T Cells With IgD-Fc-Ig and IgD

CD4^+^ T cells of healthy controls were cultured at 2 × 10^7^ cells/mL in RMPI 1640 supplemented with 5% fetal bovine serum (FBS). Human IgD protein (FITC-IgD) was labeled with FITC fluorescent labeling kit (DOJINDO LABORATORIES). CD4^+^ T cells were incubated with various concentrations of IgD-Fc-Ig (0.03, 0.1, 0.3, 1, 3, 10, 30 μg/mL) and FITC labeled human IgD (10 μg/mL) at 37 for 2 h. Bound IgD on CD4^+^ T cells were detected by flow cytometry (Beckman Coulter), and the mean fluorescence intensity (MFI) of IgD binding to IgDR was calculated.

### Human Cell Isolation and Viability Detection

PBMCs were isolated from blood samples taken from healthy controls and RA patients by Ficoll gradient centrifugation. CD4^+^ T cells from PBMCs were isolated by using CD4^+^ magnetic cell sorting (MACS) columns (Miltenyi Biotech) as previously described (15). Purity was determined to be higher than 95%. Cell activity was observed using Trypan blue staining (98% viable). Cells were cultured at 2 × 10^6^ cells/mL in RMPI 1640 supplemented with 5% FBS. Save for the control group, cells were stimulated with 3 μg/mL of IgD or anti-CD3/CD28 (0.4 μg/mL) in combination with different concentrations of IgD-Fc-Ig fusion protein (1, 3, and 10 μg/mL) for 48 h at 37°C. A Lck inhibitor A770041 group was used as a positive control, while the IgG_1_-Fc protein treatment group was used as a negative control. After treatment, a Cell Counting Kit-8 (CCK-8) was used to evaluate cell proliferation using stimulation indices according to published protocols (17, 19).

### Real-time Quantitative PCR Analysis

Following treatment of cell cultures with IgD and varying concentrations of IgD-Fc-Ig for 48 h, the total RNA from PBMCs was extracted using TRIzol Reagent (Invitrogen) and reverse-transcribed into cDNA. Glyceraldehyde-3-phosphate dehydrogenase (*GAPDH*) was used as an internal control gene for mRNA expression. *TBET, GATA3, ROR*γ*t*, and *FOXP3* genes were synthesized using specific primer sequences (Sangon Biotech, China). Transcription levels of target genes were analyzed by real-time quantitative PCR (qPCR) using an ABI 7500 (Applied Biosystems) and SYBR Green Master Mix (Vazyme). The novel primer sequences of *TBET, GATA3, ROR*γ*t*, and *FOXP3* genes are as follows:

*TBET*-Forward 5′-GCAGCACCGCTACTTCTACC-3′
*TBET*-Reverse 5′-GTAGGCGTAGGCTCCAAGG-3′
*GATA3*-Forward 5′-AGAGCGTGGCCTGGAGAC-3′
*GATA3*-Reverse 5′-CTCCCACAGTCAAGGAAACC-3′
*ROR*γ*t*-Forward 5′-AAATCTGTGGGGACAAGTCG-3′
*ROR*γ*t*-Reverse 5′-CTGACGGGTGCAGGAGTAG-3′
*FOXP3*-Forward 5′-GACAGTTTCCCACAAGCCAG-3′
*FOXP3*-Reverse 5′-TGGTGAAGTGGACTGACAGA-3′
*GADPH*-Forward 5′-CAGGAGGCATTGCTGATGAT-3′
*GADPH*-Reverse 5′-GAAGGCTGGGGCTCATTT-3′.

### Induction and Treatment of CIA Mouse

Eight-week-old male DBA/1 mice (18 ± 2 g) (Certificate No: 2016-0006) were maintained in a specific pathogen-free animal laboratory at Anhui Medical University. Type II collagen sample was dissolved in acetic acid (0.1 mol/L) at 2 mg/mL and incubated overnight at 4°C. The sample was then emulsified with an equal volume of complete Freund's adjuvant. Arthritis was induced in DBA mice by intradermal injection of 0.15 mL CII emulsion into the base of the tail followed by a booster injection of 0.1 mL emulsion on day 21. Mice were divided into nine treatment groups: Normal, CIA, IgD-Fc-Ig (1.625, 3.25, 6.5, and 13 mg/kg), IgG_1_-Fc (13 mg/kg), rhTNFR:Fc (4.5 mg/kg), and anti-mouse IgD antibody (2 mg/kg). After the onset of arthritis at day 30, mice were treated with IgD-Fc-Ig, IgG_1_-Fc, or rhTNFR:Fc through tail intravenous injection (twice weekly, for 4 weeks), and anti-IgD antibody (once daily, for 3 days) (20). rhTNFR:Fc and anti-IgD antibody treatment groups were used as positive controls (21, 22). Mice were sacrificed on day 54, and spleen and ankle joints were collected for histopathological analysis (22). Mice were evaluated every 2–3 days using arthritis index and swollen joint count. Inflammation of paws was graded on a scale of 0 to 4: 0, paws with no swelling or focal redness; (1) paws with swollen joints; (2) paws with mild swelling of ankle or wrist joints; (3) paws with severe inflammation; (4) paws with deformity or ankylosis. Each paw was graded, and the four scores were totaled so that the maximum possible score per mice was 16. Each paw contains five phalanx joints and one ankle or wrist joint; thus, the maximum swollen joint count for each mouse was 24.

Histopathology changes in joint tissue were evaluated by inflammation, pannus, bone erosion, cellular infiltration, and synovial hyperplasia. The grading scheme consisted of ordinal categories ranging from 0 (no effect) to 3 (severe effect). The spleens were evaluated by examining the total number of germinal centers (GCs), lymphoid follicles, cellularity of periarteriolar lymphoid sheaths (PLAS), marginal zone, and red pulp. The grading scheme consisted of ordinal categories ranging from 0 (no effect) to 4 (severe effect).

### Mice Thymocyte Viability Detection

Mice thymi were pressed and washed through a 200-gauge mesh. The thymocytes were cultured at 5 × 10^6^ cells/mL in RMPI 1640 supplemented with 5% FBS and stimulated with 5 μg/mL Concanavalin A (ConA) (23) for 48 h at 37°C. Mice thymocyte viability was detected by CCK-8 as previously described (23).

### Mice Plasma sIgD, Cytokines, and Chemokines Detection

Blood was collected in anticoagulant tubes containing heparin sodium on day 54 and then centrifuged at 2,500 rpm for 10 min to isolate the plasma. Mice plasma levels of sIgD were quantified by ELISA. The fluorescence signal value of interleukin (IL)-1α, IL-15, monocyte chemoattractant protein 5 (MCP-5), and macrophage colony-stimulating factor (MCSF) was measured using Quantibody Mouse Inflammation Array 1 (Raybiotech, Norcross, USA). The signals were captured using a GenePix 4000B Laser Scanner (Bio-Rad Laboratories) and extraction with GenePix Pro 6.0 Microarray Analysis Software.

### Flow Cytometry Analysis

Cells from human and mice samples were stained with fluorescently labeled mAbs against surface molecules (CD69 and CD154). For intracellular transcription factor staining, cells were fixed and permeabilized using a cell fixation/permeabilization kit (Invitrogen, Thermo Fisher Scientific) after staining for the surface markers CD4 and CD25. Cells were then incubated with IL-4, IFN-γ, IL-17, and FOXP3 antibodies, respectively. Th17 cells were stimulated with cell stimulation cocktail (500×) (eBioscience). All data were collected using flow cytometry (Beckman Coulter) and analyzed with CytoExpert (23).

### Western Blot Analysis

For the *in vitro* study, PBMCs from RA patients were collected after incubating with IgD and IgD-Fc-Ig for 48 h. Cells were lysed in lysis buffer supplemented with protease inhibitors and phosphatase inhibitors for 30 min on ice (24), whereas for the *in vivo* study, mice spleens were isolated from each group and homogenized in lysis buffer. Primary antibodies Lck (1:1,000), p-Lck (1:1,000), ZAP70 (1:1,000), p-ZAP70 (1:1,000), and β-actin (1:1,000) were then incubated at 4°C overnight, and a goat anti-rabbit secondary antibody (1:50,000) was incubated for 2 h at 37°C. The membrane was scanned using GS-700 Imaging Densitometer. The image was analyzed with Image J software.

### Statistical Analyses

Data were presented as means ± standard error (SEM). Data was checked for a normal distribution in order to decide whether to use parametric or non-parametric tests. Multigroup comparisons of the means were carried out by one-way analysis of variance (ANOVA) (SPSS 11.5 Software Products) test with *post hoc* contrasts by least significant difference test. Median group values (with standard error of the mean) were compared in patients and healthy controls using the nonparametric unpaired Mann-Whitney U test. Pearson's correlation analysis was used to examine the correlation between the parameters. *P* < 0.05 was considered statistically significant.

## Results

### Plasma IgD Levels of Healthy Controls and RA Patients

**Figure 1:**
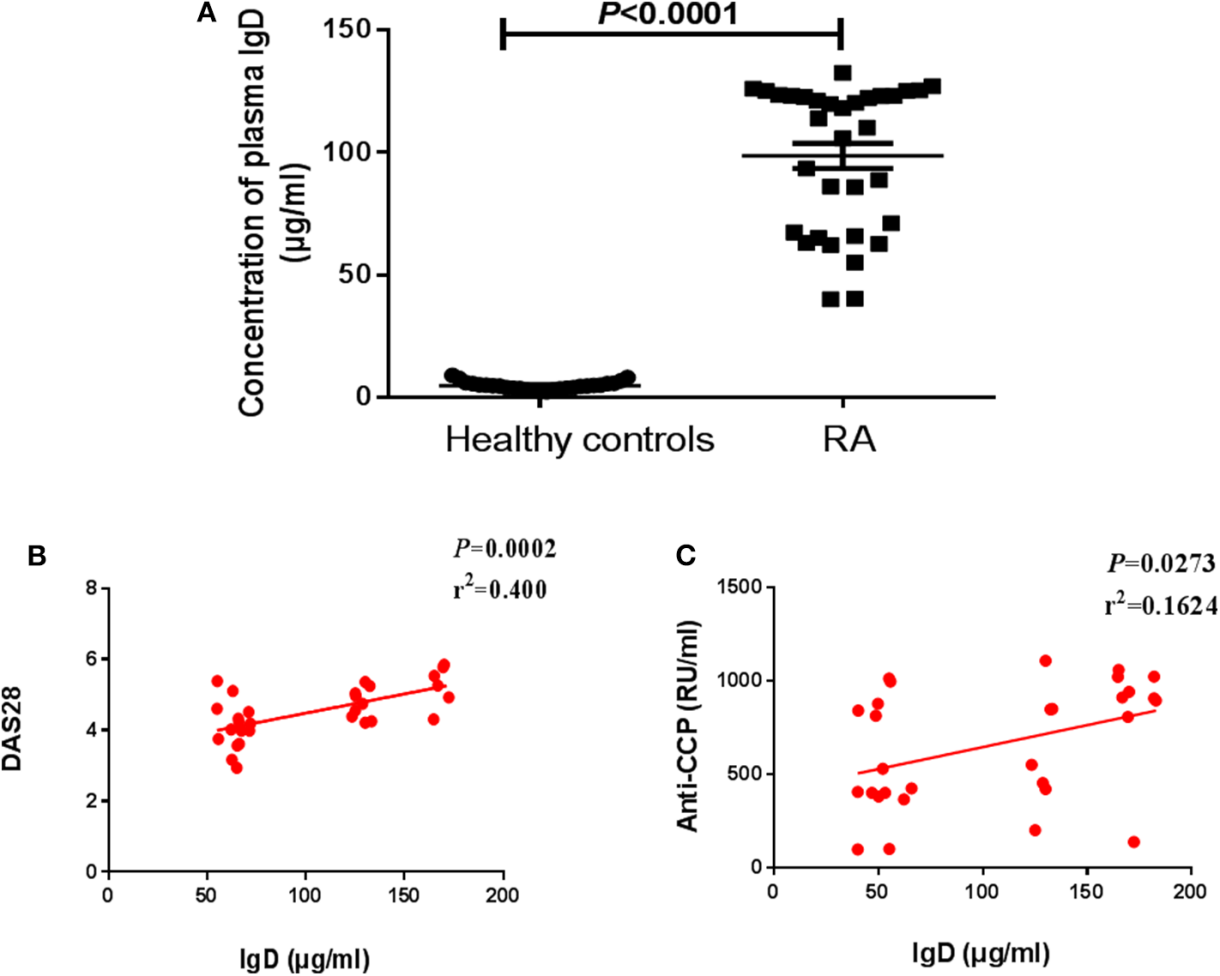
Levels of plasma sIgD in RA patients and healthy controls. **(A)** Plasma IgD levels of healthy controls (*n* = 27) and RA patients (*n* = 33). Pearson's correlation analyses were shown for the association of IgD with DAS28 scores **(B)** and anti-CCP **(C)** in RA patients.

In this study, we identified the clinical and demographic characteristics of 33 RA patients (Table 1). ELISA was used to detect the levels of plasma sIgD. To examine the correlations between the biomarkers of disease and the sIgD level in serum, Pearson's correlation analysis was performed between the DAS28 scores, anti-CCP levels of RA, and the sIgD concentrations. RA patients had higher levels of sIgD (98.55 ± 5.107 μg/mL) in their plasma compared to healthy controls (4.832 ± 0.3298 μg/mL) (*P* < 0.0001) (Figure 1A). DAS28 was used to evaluate the severity of RA. Results showed that the plasma sIgD levels were positively correlated with DAS28 score (*P* = 0.002, *r*^2^ = 0.4) and anti-CCP levels (*P* = 0.0002, *r*^2^ = 0.5975) (Figures 1B,C).

**Table 1:** Clinical characteristics of Healthy controls and RA patients.

**Characteristic**	**Healthy controls**	**RA**
Number of subjects	27	33
Female/male ratio	9:18	27:6
Age (years)	50.15 ± 1.547	57.24 ± 2.07
RF(IU/ml)	-	171.1 ± 23.8
CRP (mg/L)	-	36.71 ± 5.903
ESR (mm/h)	-	64.7 ± 4.872
Anti-CCP (RU/mL)	-	683.9 ± 55.04

*Data are means±standard Error; RF, rheumatoid factor; CRP, C-reactive protein; ESR, erythrocyte sedimentation rate; anti-CCP, anti-cyclic citrullinated peptide*.

### Competitive Binding Ability of IgD-Fc-Ig to IgDR

**Figure 2:**
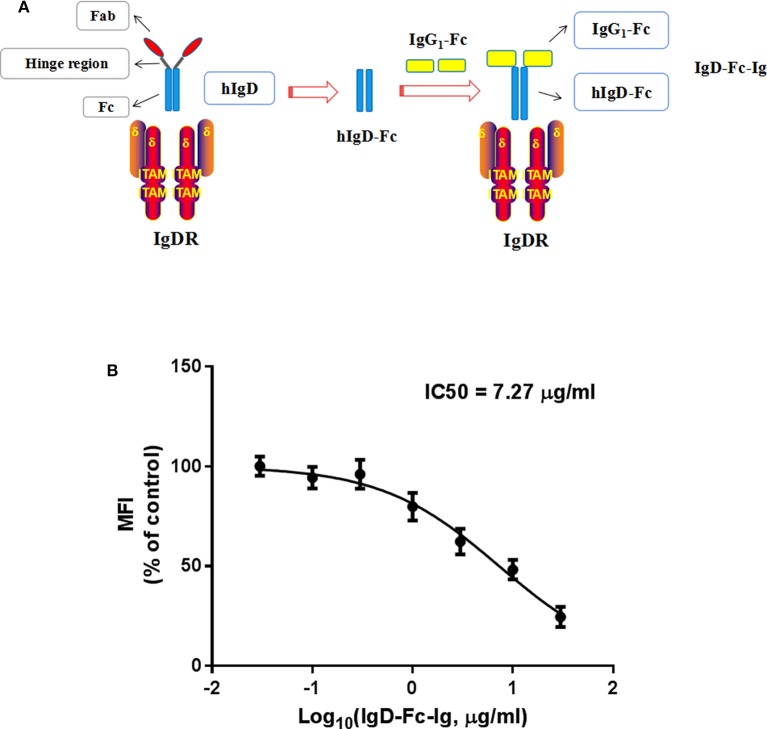
The competitive binding assay of IgD-Fc-Ig to IgDR. **(A)** The schematic diagram of IgD-Fc-Ig structure. **(B)** The competitive binding curve of IgD-Fc-Ig to IgDR. CD4^+^ T cells were incubated with various concentrations of IgD-Fc-Ig (0.03, 0.1, 0.3, 1, 3, 10, 30 μg/mL) and FITC labeled human IgD (10 μg/mL) at 37 for 2 h. The MFI of IgD binding to IgDR on CD4^+^ T cells was calculated.

In order to block IgD-IgDR binding, IgD-Fc-Ig fusion protein was synthesized as a pseudoligand for IgD (Figure 2A). Flow cytometry was used for determination the competitive binding ability of IgD-Fc-Ig to IgDR. Data showed that with the increase of IgD-Fc-Ig concentration, the fluorescence intensity of FITC on CD4^+^ T cell surface gradually decreased, suggesting that the strength of IgD binding to IgDR was reduced in a concentration-dependent manner by IgD-Fc-Ig. IgD-Fc-Ig (IC_50_ = 7.27 μg/mL) exhibited a similar binding affinity as IgD for IgDR, and competitively bind to IgDR (Figure 2B).

### Effects of IgD-Fc-Ig on the Function of Human PBMCs and T Cells Induced by IgD

**Figure 3:**
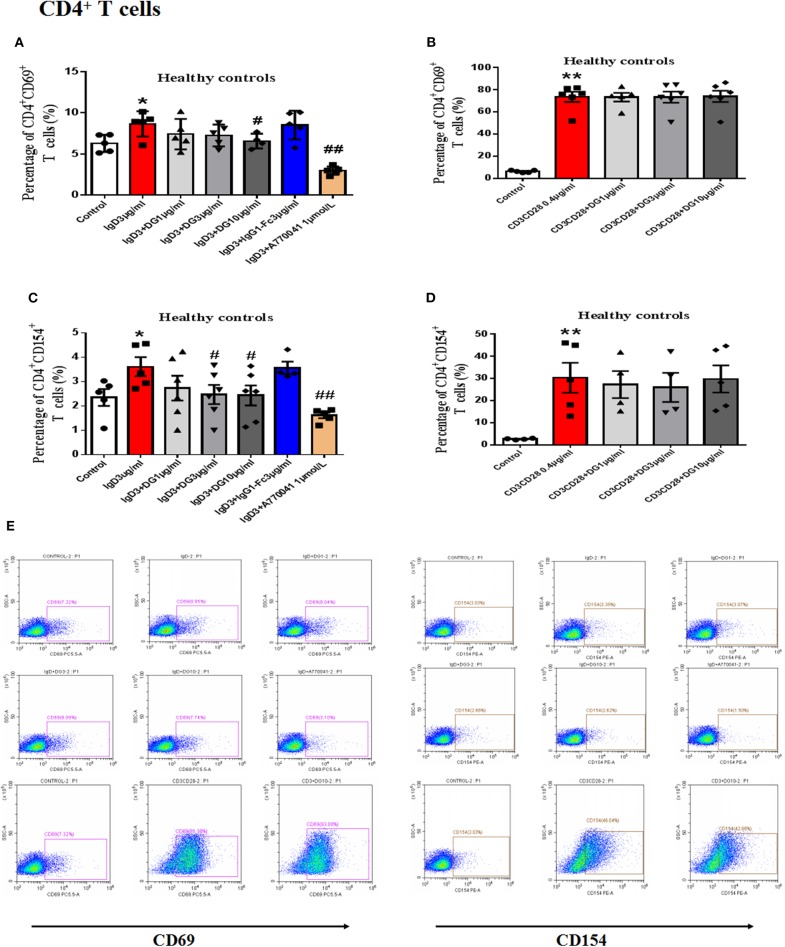
Effects of IgD-Fc-Ig (DG) on the functions of T cells in healthy controls induced by IgD. T cells were incubated with IgD (3 μg/mL) and different concentrations of IgD-Fc-Ig (1, 3, and 10 μg/mL) for 48 h. **(A)** Percentage of CD4^+^CD69^+^ T cells treated with IgD-Fc-Ig. **(B)** Percentage of CD4^+^CD69^+^ T cells treated with anti-CD3CD28 antibodies. **(C)** Percentage of CD4^+^CD154^+^ T cells treated with IgD-Fc-Ig. **(D)** Percentage of CD4^+^CD154^+^ T cells treated with anti-CD3CD28 antibodies. **(E)** Representative flow cytometry dot plot from each group. Data were expressed as mean±SEM (n=3-5). ^*^P < 0.05 and ^**^P < 0.01 vs. control, ^#^*P* < 0.05 and ^##^*P* < 0.01 vs. IgD (3 μg/mL) group.

**Figure 4:**
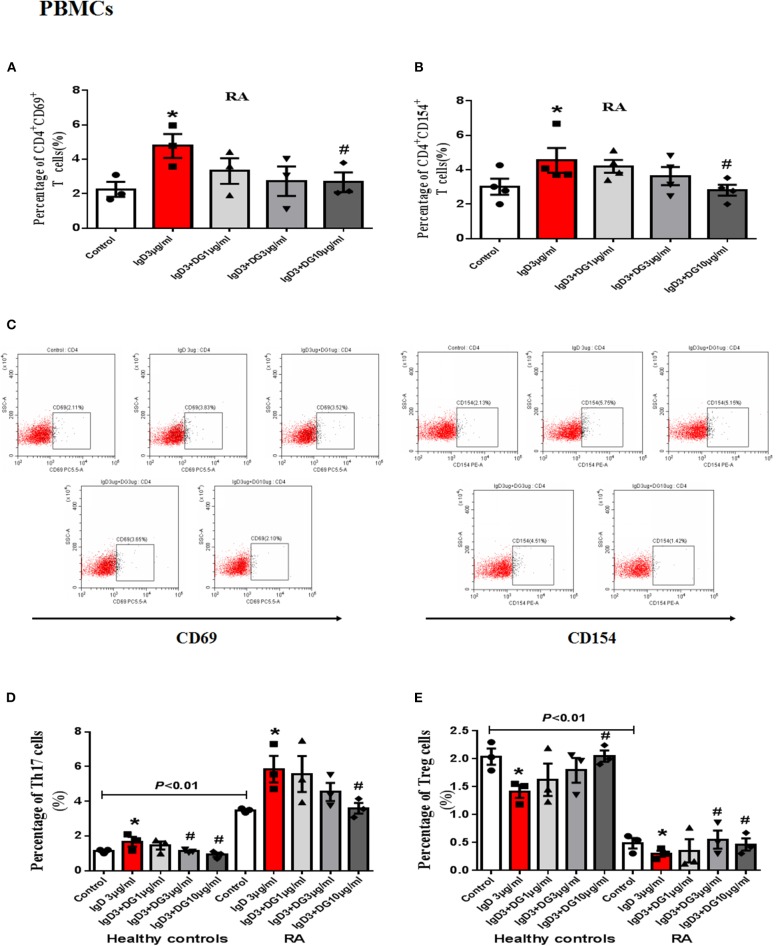
Effects of IgD-Fc-Ig (DG) on the functions of PBMCs induced by IgD. PBMCs of RA patients were incubated with IgD (3 μg/mL) and different concentrations of IgD-Fc-Ig (1, 3, and 10 μg/mL) for 48h. Percentage of CD4^+^CD69^+^ and CD4^+^CD154^+^ T cells **(A,B)** in RA patients treated with IgD-Fc-Ig. **(C)** The flow cytometry graphs are shown. Percentage of Th17 cells (CD4^+^IL17^+^ T cells) and Treg cells (CD4^+^CD25^+^FoxP3^+^ T cells) in RA patients and healthy controls treated with IgD-Fc-Ig **(D, E)**. Data were expressed as mean±SEM (*n* = 3–5). ^*^P < 0.05 vs. control, ^#^*P* < 0.05 vs. IgD (3 μg/mL) group.

*In vitro* study, IgD could promote the proliferation of PBMCs and CD4^+^ T cells, IgD-Fc-Ig could significantly inhibit IgD-induced PBMCs, and CD4^+^ T cell proliferation (*P* < 0.05) (Supplementary Figure 1). We also observed the effects of IgD-Fc-Ig on CD4^+^CD154^+^ and CD4^+^CD69^+^ T cells (activated T cells) in healthy controls and RA patients. IgD-Fc-Ig could significantly decrease the percentage of CD4^+^CD154^+^ and CD4^+^CD69^+^ T cells following IgD stimulation (*P* < 0.05) (Figures 3A,C,E, 4A–C). In healthy controls, IgD-Fc-Ig had no significant effect on activated T cells following anti-CD3CD28 stimulation (Figures 3B,D), while it downregulated the IgD-induced Th17 cells in RA patients and upregulated the Treg cells stimulated by IgD (*P* < 0.05) (Figures 4D,E). The results of healthy controls were consistent with RA patients. We did not observe any significant effects following treatment with IgD or IgD-Fc-Ig on the percentage of Th1 and Th2 cells in healthy controls or RA patients. Simultaneously, in RA patients and healthy controls, IgD increased the mRNA expression of *ROR*γ*t* and decreased the expression of *FOXP3*. As expected, treatment with IgD-Fc-Ig decreased the mRNA expression level of *ROR*γ*t* and increased the mRNA level of *FOXP3* (*P* < 0.01). No significant difference was observed in the mRNA expression of *TBET* and *GATA* in the control, IgD, or IgD-Fc-Ig-treated groups (Supplementary Figure 2).

### Effects of IgD-Fc-Ig on Protein Expression of p-Lck and p-ZAP70 in RA PBMCs

After PBMCs were incubated with IgD for 48 h in RA patients, the protein expression levels of p-Lck and p-ZAP70 were found to increase by 1.17- and 1.47-fold, respectively, compared to the control. IgD-Fc-Ig treatment significantly reduced the expression of p-Lck and p-ZAP70 compared to that of the control (*P* < 0.01) (**Figures 9A–C**).

### Anti-inflammatory Effects of IgD-Fc-Ig on CIA Mice

**Figure 5:**
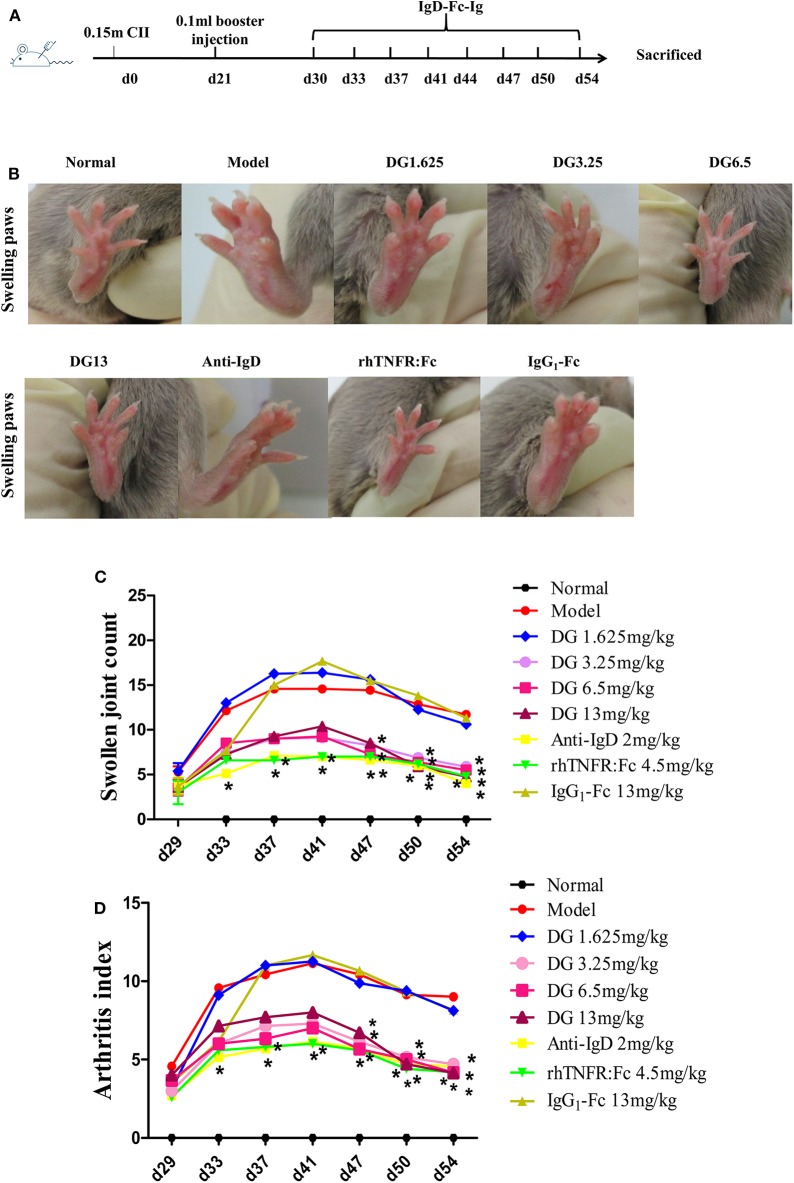
Effects of IgD-Fc-Ig (DG) on swollen joint count and arthritis index of CIA mice. Anti-inflammatory effects of IgD-Fc-Ig (DG) on CIA mice. DBA/1 male mice were immunized with CII and FCA on D0 and D21. Then the mice were treated with IgD-Fc-Ig, IgG_1_-Fc, rhTNFR:Fc, and anti-mouse IgD antibody by tail intravenous administration. **(A)** The time points of IgD-Ig-Fc fusion protein administrated. **(B–D)** Effects of IgD-Fc-Ig on swollen joint count and arthritis index of CIA mice. ^*^P < 0.05 vs. Model.

**Figure 6:**
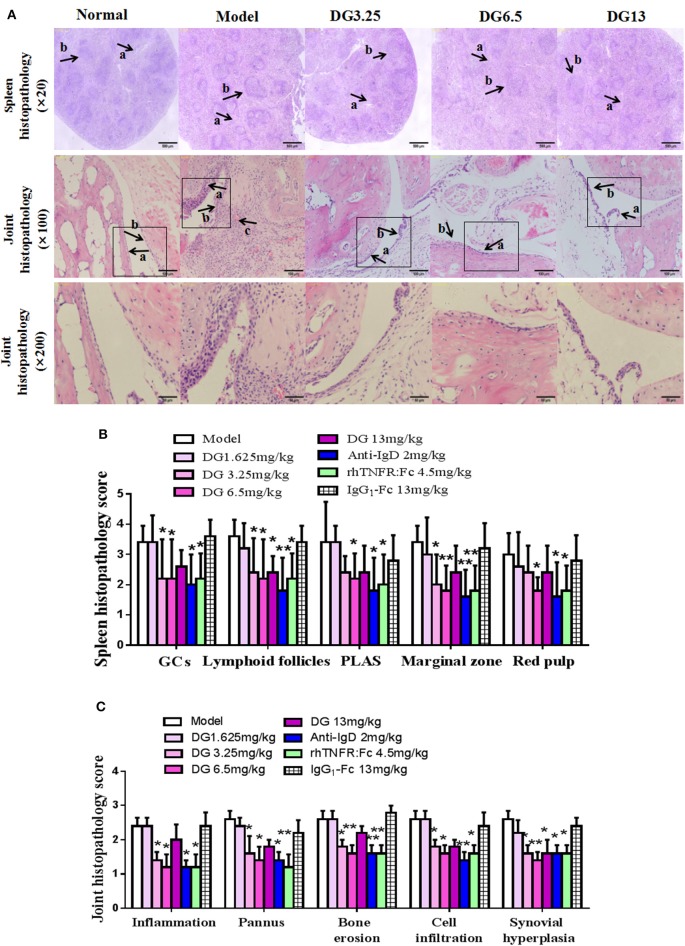
Effects of IgD-Fc-Ig (DG) on ankle joints and spleens histopathology of CIA mice. **(A)** A photomicrograph of spleen histopathology showing red pulp congestion (arrow a), and white pulp proliferation (arrow b). Original magnification ×20. A photomicrograph of ankle joint histopathology showing synoviocyte hyperplasia (arrow a), cellular infiltration (arrow b), articular cartilage destruction and pannus (arrow c). Original magnification ×100 and ×200. **(B)** Histological appearance was scored for the presence of the total number of GCs, lymphoid follicles, PALS, marginal zone the red pulp in mice spleens. **(C)** Histological appearance was scored for the presence of inflammation, pannus, bone erosion, cell infiltration, and synovial hyperplasia in mice ankle joints. Data were expressed as mean±SEM (For each group 5–9 mice). ^*^P < 0.05 and ^**^P < 0.01 vs. Model.

Secondary joint inflammation was apparent on approximately day 29 following the first immunization. CIA mice developed typical manifestations of severe arthritis and showed front and hind paw swelling and redness (Figure 5A). Administration of IgD-Fc-Ig (3.25, 6.5, and 13 mg/kg) significantly alleviated these abnormalities in varying degrees (*P* < 0.05) (Figures 5B–D). Results from the histopathology of ankle joints in CIA mice showed synoviocyte proliferation, pannus formation, damaged articular cartilage, as well as inflammatory infiltration and bone erosion, the severity of which was significantly attenuated following administration of IgD-Fc-Ig (3.25, 6.5, and 13 mg/kg) (*P* < 0.05) (Figures 6A,C). Similarly, the histopathology of the spleen, which was characterized by white pulp proliferation, emergence of germinal centers (GCs), and infiltration by inflammatory cells, was significantly alleviated by IgD-Fc-Ig (3.25, 6.5, and 13 mg/kg) treatment (*P* < 0.05) (Figures 6A,B). Anti-IgD antibody and rhTNFR:Fc treatment exhibited similar effects to that observed with IgD-Fc-Ig treatment (Figures 5, 6).

### Effects of IgD-Fc-Ig on the T Cell Function of CIA Mice

**Figure 7:**
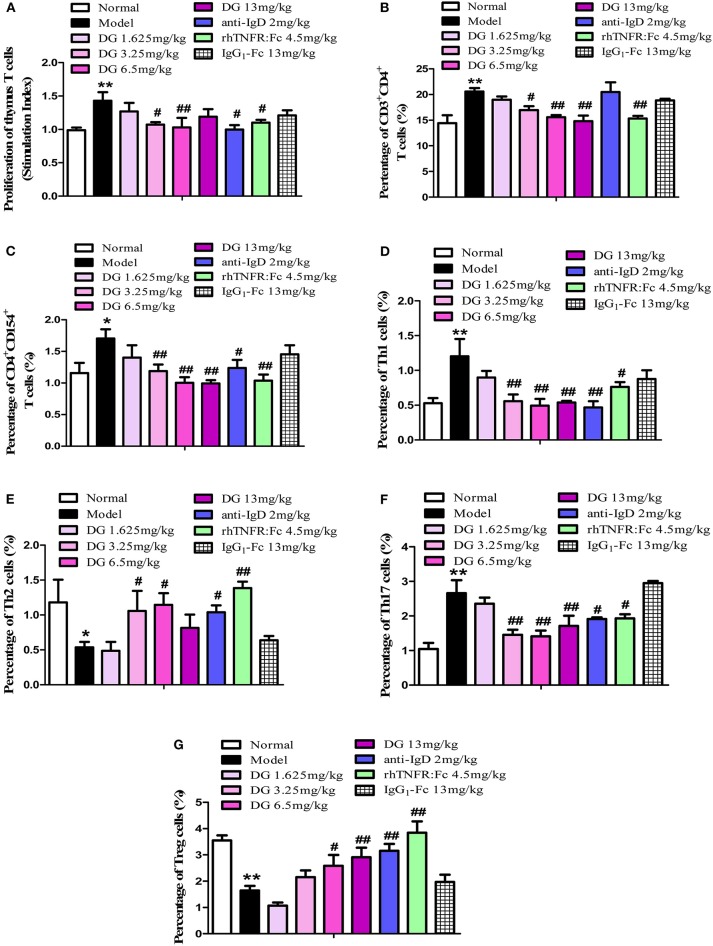
Effects of IgD-Fc-Ig (DG) on the T cells functions of CIA mice. **(A)** Effects of IgD-Fc-Ig on the proliferation of thymus T cells. Effects of IgD-Fc-Ig on the percentage of CD3^+^CD4^+^ **(B)**, CD4^+^CD154^+^ **(C)**, Th1 (CD4^+^ IFNγ^+^) **(D)**, Th2 (CD4^+^IL4^+^) **(E)**, Th17 (CD4^+^IL17^+^) **(F)**, and Treg (CD4^+^CD25^+^Foxp3^+^) **(G)**. Data were expressed as mean±SEM (For each group 4–6 mice). ^*^P < 0.05 and ^**^P < 0.01 vs. Normal, ^#^*P* < 0.05 and ^##^*P* < 0.01 vs. Model.

The proliferation of thymocytes was detected by CCK8. Our results show that IgD-Fc-Ig treated mice prevented the proliferation of ConA-stimulated thymocytes in CIA mice (*P* < 0.01) (Figure 7A). In comparison with the control, CIA mice exhibited a higher percentage of Th cells, specifically CD4^+^CD154^+^ T cells, Th1, and Th17. After administration of IgD-Fc-Ig (3.25, 6.5, and 13mg/kg), the percentage of these cells significantly decreased (*P* < 0.01) (Figures 7B–D,F). Flow cytometry results also revealed that the levels of Th2 and Treg cells were significantly reduced in CIA mice. IgD-Fc-Ig treatment of these CIA mice significantly elevated the levels of Th2 (*P* < 0.05) and Tregs (*P* < 0.01) (Figures 7E,G).

### Effects of IgD-Fc-Ig on IgD Levels, and Cytokine and Chemokine Function in CIA Mice

**Figure 8:**
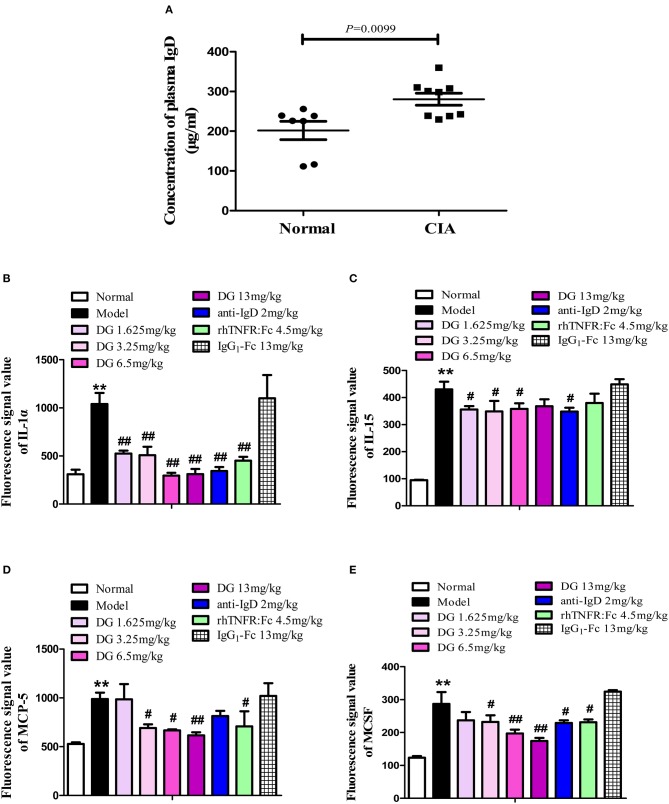
Effects of IgD-Fc-Ig (DG) on IgD levels, cytokines, and chemokines function of CIA mice. Effects of IgD-Fc-Ig on the plasma levels of sIgD **(A)** and secretion of IL-1α **(B)**, IL-15 **(C)**, MCP-5 **(D)**, and MCSF **(E)**. Data were expressed as mean±SEM (For each group 4–6 mice). ^**^P < 0.01 v*s*. Normal, ^#^*P* < 0.05 and ^##^*P* < 0.01 vs. Model.

Mice plasma levels of sIgD were detected by ELISA. We found that IgD levels in CIA mice (280.7 ± 15.00 μg/mL) were significantly higher than in the control (201.9 ± 23.01 μg/mL) (*P* = 0.0099) (Figure 8A). Antibody microarray results showed that, compared with the normal group, the levels of plasma IL-1α, IL-15, MCP-5, and MCSF were significantly elevated in CIA mice. With the administration of IgD-Fc-Ig, IL-1α, and IL-15 levels were significantly reduced in CIA mice (Figures 8B,C), as was the ameliorating MCP-5 and MCSF production (Figures 8D,E).

### Effects of IgD-Fc-Ig on the Protein Expression of Lck, p-Lck, ZAP70, and p-ZAP70 in CIA Mice

**Figure 9:**
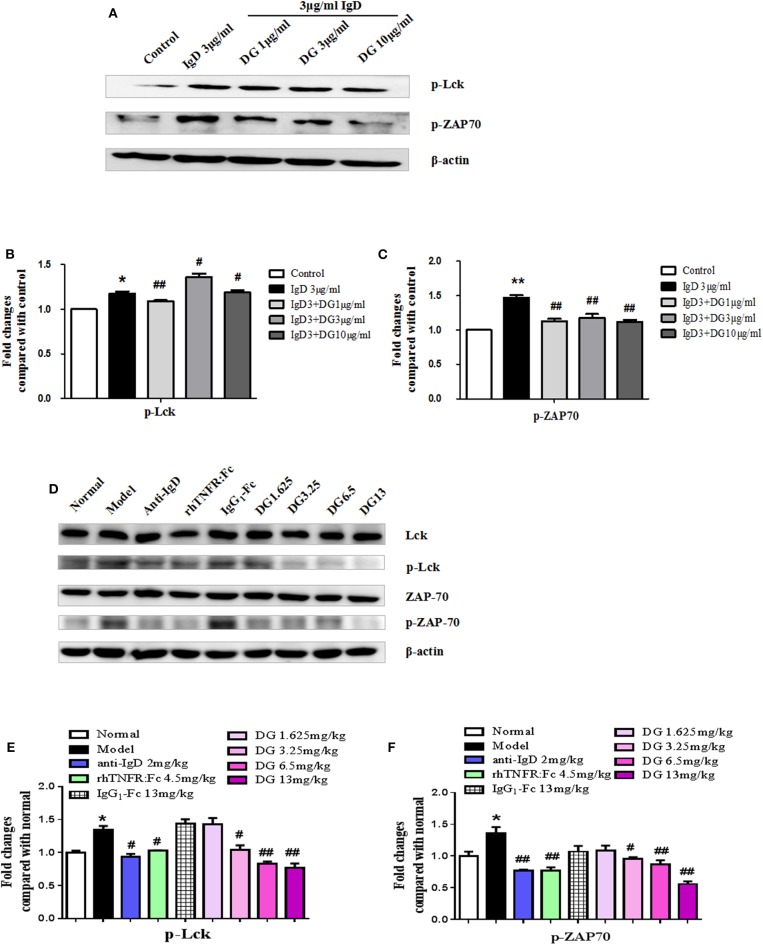
Effects of IgD-Fc-Ig (DG) on the protein expression of Lck, p-Lck, ZAP70 and p-ZAP70. **(A–C)** Western blot analysis of p-Lck and p-ZAP70 expression in PBMCs of RA patients, PBMCs were treated with IgD (3 μg/mL) and IgD-Fc-Ig (1, 3, 10 μg/mL) for 48h and lysed. **(D–F)** Western blot analysis of Lck, p-Lck, ZAP70 and p-ZAP70 expression in CIA mice. Mice spleens from each group were isolated and homogenized in lysis buffer. Data were expressed as mean±SEM (*n* = 3). ^*^P < 0.05 and ^**^P < 0.01 vs. Control and Normal, ^#^*P* < 0.05 and ^##^*P* < 0.01 vs. IgD (3 μg/mL) group and Model.

No significant differences were observed in the total protein expression of Lck and ZAP70 among any of the study groups; however, the expression of p-Lck in CIA mice was upregulated 1.34-fold and p-ZAP70 protein expression was increased 1.36-fold compared with the control mice. Additionally, IgD-Fc-Ig-treated groups exhibited lower p-Lck expression compared to the model group (*P* < 0.01) (Figures 9D–F).

## Discussion

The association between detectable sIgD and biomarkers including disease activity score in 28 joints (DAS28) and anti-CCP in patients with RA were observed. DAS28 score is a comprehensive indicator for evaluating the disease activity of RA, including number of swollen and tender joints and laboratory indicators. It is currently the best comprehensive indicator in clinical practice for monitoring the disease activity of RA and evaluating the effectiveness of drug therapy (25, 26). Anti-CCP antibody can be used as predictor of disease course and treatment outcome in RA (27). Our data indicated that sIgD levels were closely associated with severity of RA. We set the cut-off value of plasma IgD level as 80 μg/mL (28), and founded that 23 out of the 33 RA patients were higher than the cut-off value. Our previous work showed that *in vitro* IgD effectively promotes PBMC proliferation in RA patients (17) and facilitates CD4^+^ T cell proliferation and activation in healthy controls (29). Nguyen et al. have reported that anti-IgD treatment modulates the innate and adaptive cytokine responses in both mice and humans and alleviated collagen-induced arthritis in mouse (20). Taken together, IgD may participate in the pathogenesis of RA.

We have investigated that IgDR expression on T cells, induced by IgD, was higher than on B cells both in RA patients and healthy controls (17). This implies that IgD-IgDR interactions on T cells may be involved in the progression of RA and could be a promising target for RA treatment. However, current IgD antibodies used in therapy have several limitations, such as short half-life and high immunogenicity. The immunogenicity of anti-IgD antibodies may induce humoral immune responses in patients causing the formation of anti-drug antibodies (30, 31). Anti-IgD treatment showed effectiveness when administered at the early onset of RA (20). In this study, we synthesized human IgD-Fc protein, which results in lower immunogenicity, to selectively block IgD-IgDR interaction and to inhibit excessive IgD activation. The IgG_1_ Fc region can delay lysosomal degradation of immunoglobulins by cycling them back into the blood and prolonging plasma half-life due to its ability to bind to FcR (32).We then synthesized and purified a novel biological agent, IgD-Fc-Ig fusion protein, by combining a IgG_1_-Fc domain to IgD-Fc, aiming to bind to IgDR and thereby prevent excessive IgD activation.

To mimic the blood environment in RA, we used IgD or anti-CD3/CD28 antibodies to activate T cells *in vitro*. After IgD treatment, IgD-Fc-Ig selectively inhibited the proliferation of PBMCs in RA patients and CD4^+^ T cells in healthy controls, as we had hypothesized. IgD-Fc-Ig had no significant effect on anti-CD3/CD28 antibody-stimulated cells. Activated T cells, together with high expression of CD69 and CD154 (CD40L), play vital roles in RA-associated inflammation by producing inflammatory cytokines, which induce synovium inflammation and immune responses (33, 34). We previously demonstrated that IgD increases the percentage of CD69^+^ and CD154^+^ cells, which might contribute to the elevated IgDR expression and Lck tyrosine phosphorylation (19). The specific blocking of the activated T cells induced by IgD suggests that IgD-Fc-Ig may prevent T cell over-proliferation and activation through selectively targeting IgDR.

A functional imbalance in Th1/Th2 and Th17/Treg cells may be responsible for the development and progression of RA (35). Additionally, overall depletion of Tregs and an increase in Th17 cell levels in the peripheral blood and target organs can be detected in RA patients (36–38). Hence, regulation and recovery of T cell subset balance is a critical strategy for RA treatment (39). We previously reported that IgD broke the balance between Th17 and Treg cells by increasing the ratio of Th17/Treg cells and influencing the *ROR*γ*t* and *FOXP3* mRNA expression in healthy controls (29). This study showed that the ratio of Th17/Treg cells in RA patients was significantly higher than in healthy controls, and IgD had a more significant impact on PBMCs from RA patients. After treatment with IgD-Fc-Ig *in vitro*, the imbalance of Th17/Treg was restored.

We further established a CIA mouse model (40, 41), which is a typical animal model of RA, to assess the effect of IgD-Fc-Ig *in vivo*. Consistent with clinical findings, CIA mice presented a higher level of sIgD in plasma, while inflammatory indicators of CIA mice were significantly alleviated after IgD-Fc-Ig treatment, suggesting the essential role of IgD in the development of CIA. Although significant therapeutic effects of IgD-Fc-Ig treatment were first noticed when the dosage was increased to 3.25 mg/kg, the optimal dosage of IgD-Fc-Ig was determined to be 6.5 mg/kg as no significant difference was observed between administration of 6.5 and 13 mg/kg. Anti-IgD antibody had the same therapeutic effect as IgD-Fc-Ig. The results confirmed that neutralizing excessive IgD may be a promising therapeutic strategy for RA. In CIA mice, IgD-Fc-Ig administration decreased the proliferation of thymocytes and inhibited the activation of T cells. There were parallels between human *in vitro* results and murine *in vivo* assays, suggesting that the therapeutic mechanism of IgD-Fc-Ig may be related to regulation of T cell-mediated immune responses.

The effects of IgD-Fc-Ig on inflammatory mediators was determined by analysis of specific cytokines and chemokines associated with RA. IL-1α is a well-established chronic inflammatory mediator in RA and blocking its expression results in sustained symptomatic alleviation (42, 43). IL-15 is a proinflammatory cytokine overexpressed in RA, which enhances CD4^+^ T cell proliferation in RA patients (44). MCP-5 is a potent chemoattractant for circulating monocytes to inflammatory sites (45). MCSF is the primary growth factor regulating survival, proliferation, and differentiation of hematopoietic lineage cells including monocytes, macrophages, and osteoclasts (46). The levels of IL-1α, IL-15, MCP-5, and MCSF were significantly upregulated in the plasma of CIA mice compared to the controls. However, following IgD-Fc-Ig treatment, the levels of these inflammatory mediators returned to normal.

Finally, we investigated the molecular mechanism of IgD-Fc-Ig on T-cell activity and function. Selective Lck inhibitor A770041 prevented CD4^+^ T cell proliferation and activation stimulated by IgD, confirming that Lck is a key molecular mediator of IgD-induced T cell proliferation and activation (19). Lck can be recruited to the T cell receptor (TCR)/CD3 complex and contributes to the initiation of the TCR signaling cascade, which causes the phosphorylation and activation of ZAP-70 that orchestrates T cell differentiation and proliferation (47–49). In PBMCs from RA patients, IgD-Fc-Ig significantly inhibited the phosphorylation of Lck and ZAP70 after IgD stimulation. Consistently, IgD-Fc-Ig treat groups had lower expression of p-Lck and p-ZAP70 compared with CIA mice group. Our study showed that IgD-Fc-Ig contributed to the inhibition of T cell over-activation via the IgD-IgDR-Lck axis, with high selectivity. Our data suggest two novel approaches to treat RA: first, by targeting IgD-IgDR-Lck, and second, by using IgD-Fc-Ig fusion protein, which is accompanied by high IgD expression. However, further studies focusing on the dynamic changes in sIgD levels, the interaction between T cell and other inflammatory cell types in joints, and the related molecular mechanisms are needed.

In summary, our study showed a correlation between aberrant sIgD formation and RA pathogenesis. IgD-IgDR-Lck may act via a positive feedback loop mechanism contributing to T cell activation in early stages of RA. IgD-Ig-Fc fusion protein is recommended to be administered in the early stage of RA to effectively block excessive IgD-IgDR crosslinking, which may help to alleviate RA symptoms. IgD-Fc-Ig selectively blocks IgD binding to IgDR, suppresses the abnormal proliferation and activation of T cells induced by IgD, recovers the imbalance of Th17/Treg cells, reduces inflammatory cytokine production, and downregulates the phosphorylation of Lck and ZAP70. This may explain the therapeutic effect of IgD-Fc-Ig in CIA mice.

## Data Availability Statement

All datasets generated for this study are included in the article/Supplementary Material.

## Ethics Statement

The study protocol was carried out in accordance with the Declaration of Helsinki and approved by the Ethics Committee of Anhui Medical University (No. 20160119, 20160095). The patients/participants provided their written informed consent to participate in this study.

## Author Contributions

JZ performed the experiments and wrote the manuscript. XH participated the experiments, collected the samples, and did western blot experiments. XD, XH, and WC performed experiments. LZ and YC helped to revise the manuscript. YW designed the study, participated in the experiments, and revised the manuscript. WW conceived of the study and revised the manuscript. All authors read and approved the final manuscript.

## Conflict of Interest

The authors declare that the research was conducted in the absence of any commercial or financial relationships that could be construed as a potential conflict of interest.

## Supplementary Material

Supplementary Figure 1 Effects of IgD-Fc-Ig (DG) on the proliferation of T cells in healthy controls and PBMCs in RA patients induced by IgD. Cells were incubated with IgD (3μg/ml) and different concentrations of IgD-Fc-Ig (1, 3 and 10μg/ml) for 48h. **(A)** Stimulation index of CD4^+^ T cells in healthy controls treated with IgD-Fc-Ig and IgD (3μg/ml) stimulation. **(B)** Stimulation index of PBMCs in RA patients treated with IgD-Fc-Ig and IgD (3μg/ml) stimulation. ^*^*P* < 0.05 vs. control, ^#^*P* < 0.05 vs. IgD (3μg/ml) group.

Supplementary Figure 2 Effects of IgD-Fc-Ig (DG) on the mRNA expression of T cell subsets stimulated by IgD. PBMCs were cultured with different concentrations of IgD-Fc-Ig (1, 3 and 10μg/ml) combined with IgD (3μg/ml) for 48h. QPCR was used to analyze the mRNA expression level of T cell subsets. The mRNA expression of RORγt (A1), FoxP3 (A2), GATA3 (A3) and Tbet (A4) in healthy controls treated with IgD-Fc-Ig. The mRNA expression of RORγt (B1), FoxP3 (B2), GATA3 (B3) and Tbet (B4) in RA patients treated with IgD-Fc-Ig. Data were expressed as mean±SEM (*n* = 3). ^*^*P* < 0.05 and ^**^*P* < 0.01 vs. control, ^#^*P* < 0.05 and ^##^*P* < 0.01 vs. IgD (3μg/ml) group.

## Abbreviations

Anti-CCP: anti-cyclic citrullinated peptide
CCK8: Cell Counting Kit-8
ConA: concanavalin A
CRP: C-reactive protein
DAS28: disease activity score in 28 joints
DMARDs: disease-modifying antirheumatic drugs
ESR: erythrocyte sedimentation rate
FLS: fibroblast-like synoviocytes
IgD: immunoglobulin D
IgDR: IgD receptor
IL: interleukin 1α
ITAM: immunoreceptor tyrosine-based activation motifs
LAT: family adaptor molecule linker for activation of T cells
MACS: magnetic cell separation
MCP-5: monocyte chemoattractant protein 5
MCSF: macrophage colony stimulating factor
MFI: mean fluorescence intensity
mIgD: membrane IgD
NSAIDs: nonsteroidal anti-inflammatory drugs
PBMCs: peripheral blood mononuclear cells
qPCR: real-time quantitative PCR
RA: rheumatoid arthritis
RF: rheumatoid factor
RT: room temperature
SI: stimulation index
sIgD: secreted IgD
SLE: systemic lupus erythematosus
sRANKL: soluble receptor activator of nuclear factor-κB ligand
TCR: T cell antigen receptor
TNF-α: tumor necrosis factor α.
