# Supplementary figures and images for: Regulation of T Cell Activities in Rheumatoid Arthritis by the Novel Fusion Protein IgD-Fc-Ig
Source: Front Immunol. 2020 May 15;11:755. doi: 10.3389/fimmu.2020.00755 (PMC7243948; doi:10.3389/fimmu.2020.00755)

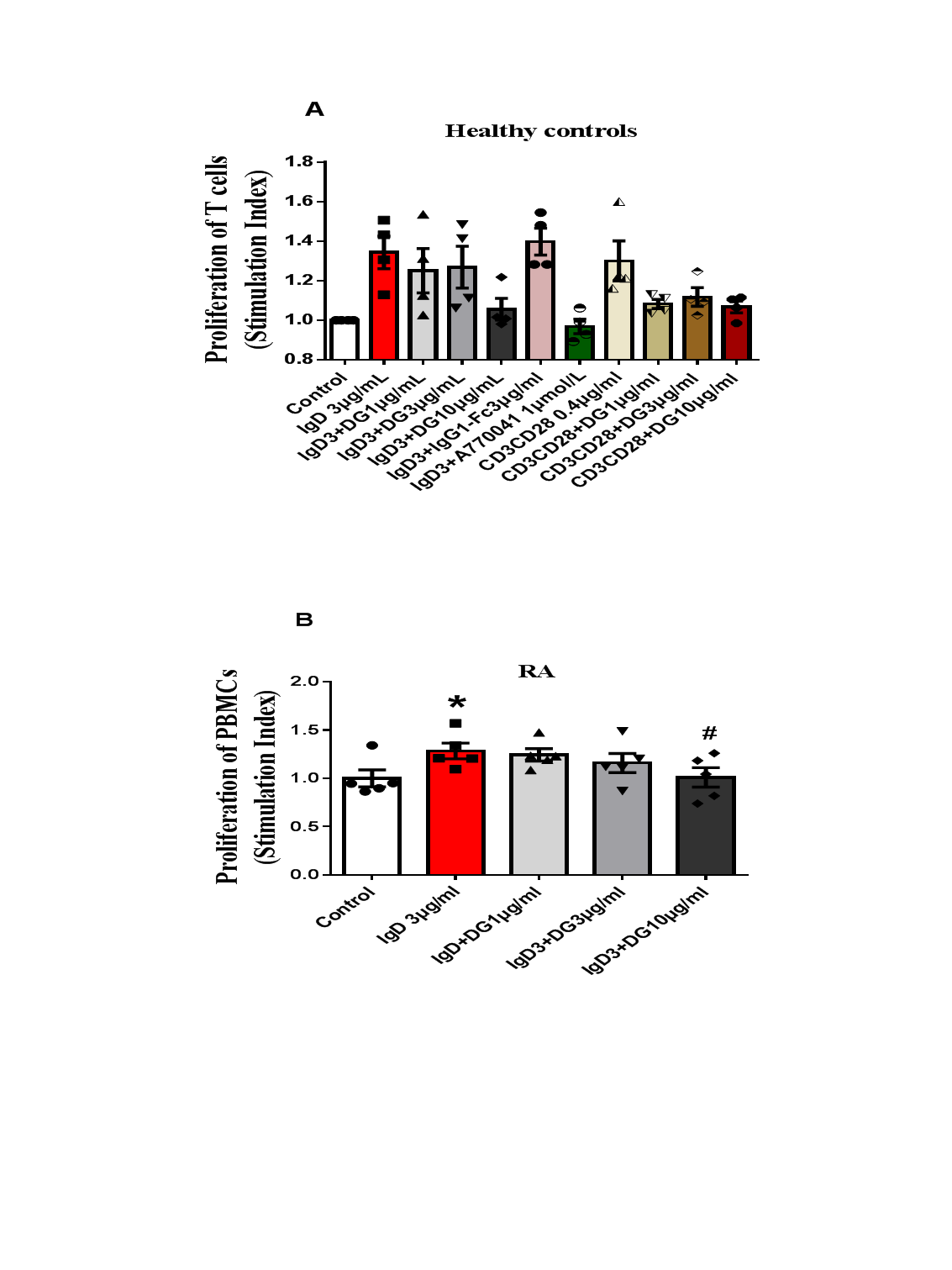

Supplement: Supplementary Figure 1 — Effects of IgD-Fc-Ig (DG) on the proliferation of T cells in healthy controls and PBMCs in RA patients induced by IgD. Cells were incubated with IgD (3μg/ml) and different concentrations of IgD-Fc-Ig (1, 3 and 10μg/ml) for 48h. (A) Stimulation index of CD4+ T cells in healthy controls treated with IgD-Fc-Ig and IgD (3μg/ml) stimulation. (B) Stimulation index of PBMCs in RA patients treated with IgD-Fc-Ig and IgD (3μg/ml) stimulation. *P < 0.05 vs. control, #P < 0.05 vs. IgD (3μg/ml) group. [file Image_1.TIF]

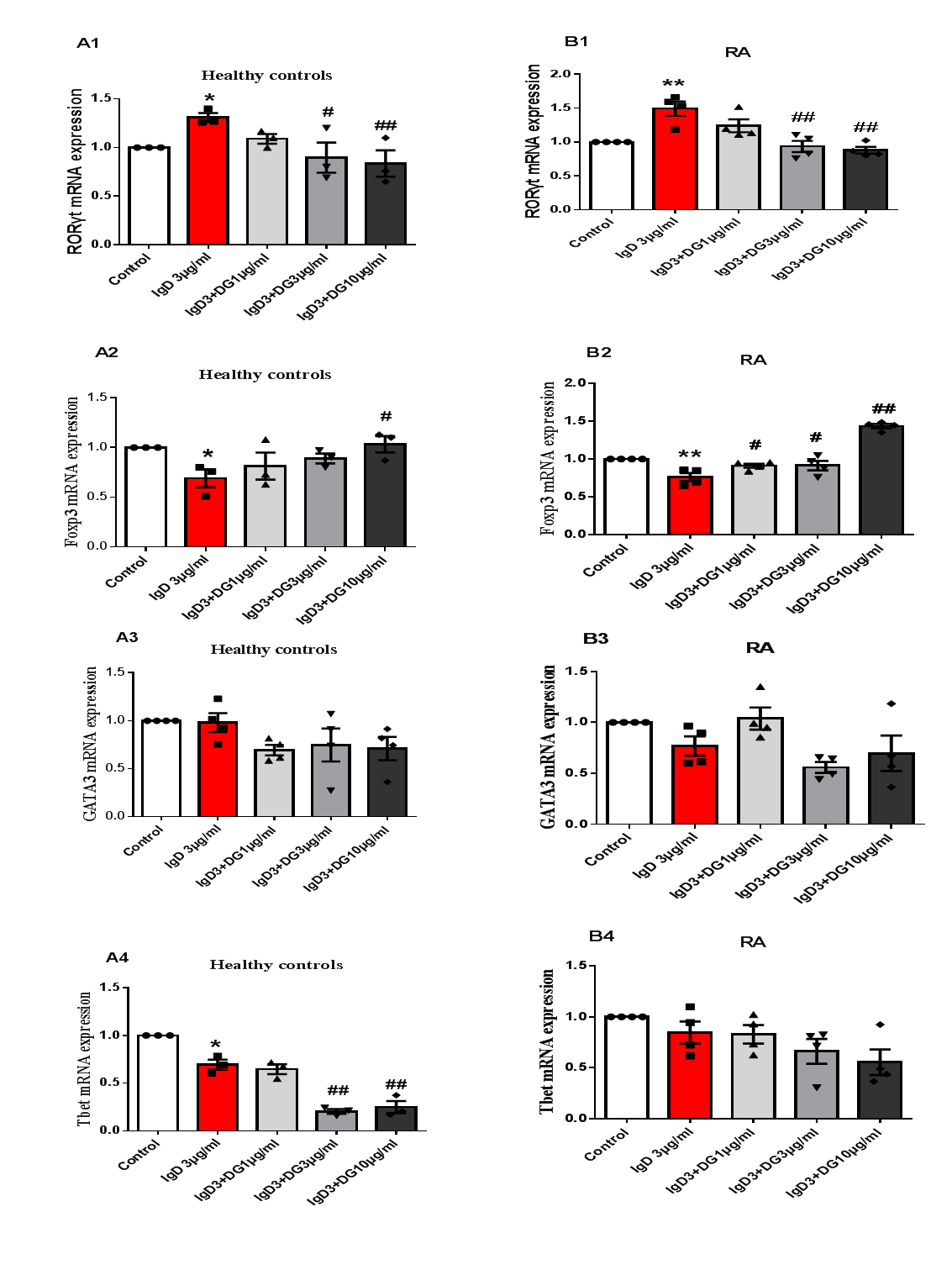

Supplement: Supplementary Figure 2 — Effects of IgD-Fc-Ig (DG) on the mRNA expression of T cell subsets stimulated by IgD. PBMCs were cultured with different concentrations of IgD-Fc-Ig (1, 3 and 10μg/ml) combined with IgD (3μg/ml) for 48h. QPCR was used to analyze the mRNA expression level of T cell subsets. The mRNA expression of RORγt (A1), FoxP3 (A2), GATA3 (A3) and Tbet (A4) in healthy controls treated with IgD-Fc-Ig. The mRNA expression of RORγt (B1), FoxP3 (B2), GATA3 (B3) and Tbet (B4) in RA patients treated with IgD-Fc-Ig. Data were expressed as mean±SEM (n = 3). *P < 0.05 and **P < 0.01 vs. control, #P < 0.05 and ##P < 0.01 vs. IgD (3μg/ml) group. [file Image_2.TIF]
